# Mutations in EPHB4 cause human venous valve aplasia

**DOI:** 10.1172/jci.insight.140952

**Published:** 2021-09-22

**Authors:** Oliver Lyons, James Walker, Christopher Seet, Mohammed Ikram, Adam Kuchta, Andrew Arnold, Magda Hernández-Vásquez, Maike Frye, Gema Vizcay-Barrena, Roland A. Fleck, Ashish S. Patel, Soundrie Padayachee, Peter Mortimer, Steve Jeffery, Siren Berland, Sahar Mansour, Pia Ostergaard, Taija Makinen, Bijan Modarai, Prakash Saha, Alberto Smith

**Affiliations:** 1 Academic Department of Vascular Surgery, Section of Vascular Risk and Surgery, School of Cardiovascular Medicine and Sciences, BHF Centre of Research Excellence, King’s College London, St. Thomas’ Hospital, London, United Kingdom.; 2 Department of Ultrasonic Angiology, Guy’s & St. Thomas’ NHS Foundation Trust, London, United Kingdom.; 3 Rudbeck Laboratory, Department of Immunology, Genetics and Pathology, Uppsala University, Sweden.; 4 Centre for Ultrastructural Imaging, King’s College London, London, United Kingdom.; 5 Molecular and Clinical Sciences Research Institute, St. George’s University of London, London, United Kingdom.; 6 Department of Medical Genetics, Haukeland University Hospital, Bergen, Norway.; 7 South West Thames Regional Genetics Service, St. George’s Hospital, London, United Kingdom.

**Keywords:** Angiogenesis, Development, Cardiovascular disease, Genetic diseases, Molecular biology

## Abstract

Venous valve (VV) failure causes chronic venous insufficiency, but the molecular regulation of valve development is poorly understood. A primary lymphatic anomaly, caused by mutations in the receptor tyrosine kinase *EPHB4*, was recently described, with these patients also presenting with venous insufficiency. Whether the venous anomalies are the result of an effect on VVs is not known. VV formation requires complex “organization” of valve-forming endothelial cells, including their reorientation perpendicular to the direction of blood flow. Using quantitative ultrasound, we identified substantial VV aplasia and deep venous reflux in patients with mutations in *EPHB4*. We used a GFP reporter in mice to study expression of its ligand, ephrinB2, and analyzed developmental phenotypes after conditional deletion of floxed *Ephb4* and *Efnb2* alleles. EphB4 and ephrinB2 expression patterns were dynamically regulated around organizing valve-forming cells. *Efnb2* deletion disrupted the normal endothelial expression patterns of the gap junction proteins connexin37 and connexin43 (both required for normal valve development) around reorientating valve-forming cells and produced deficient valve-forming cell elongation, reorientation, polarity, and proliferation. *Ephb4* was also required for valve-forming cell organization and subsequent growth of the valve leaflets. These results uncover a potentially novel cause of primary human VV aplasia.

## Graphical abstract

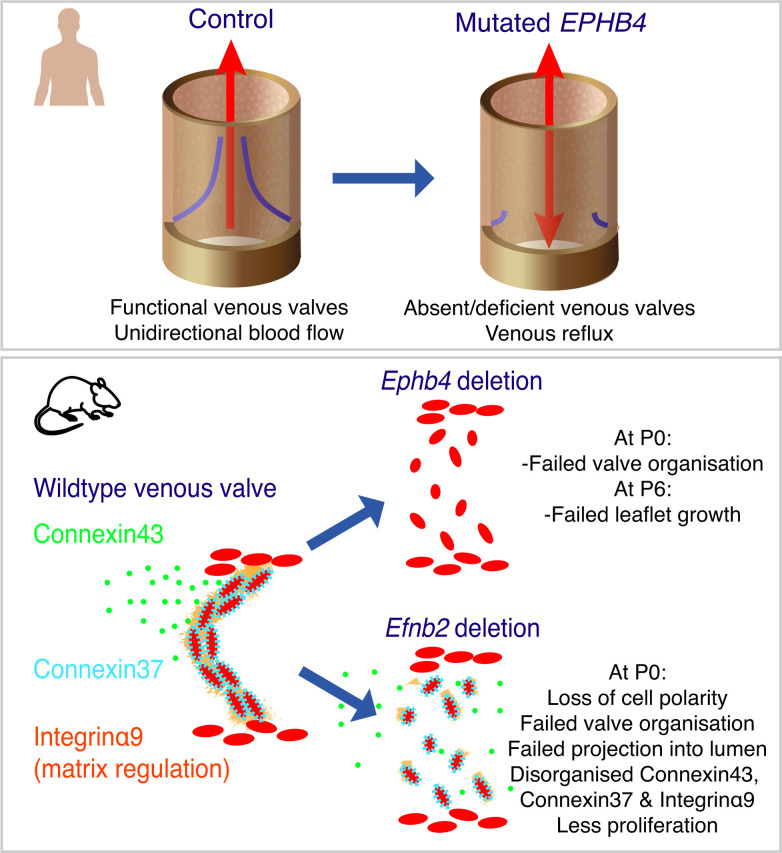

## Introduction

Unidirectional blood flow requires functional venous valves (VVs), which are widely distributed throughout human veins and venules, predominantly in vessels less than 100 μm in diameter (1). Lower limb VVs are typically bicuspid and situated just upstream of the confluence with a tributary (1, 2). Failure of these valves is the central feature of the venous reflux that is seen in up to 40% of adults (3, 4), although congenital VV aplasia has also been identified (5–8). In the lower limbs, venous reflux causes chronic venous hypertension, leading to pain, edema, hyperpigmentation, skin damage, and chronic intractable ulceration (3, 9, 10). Our understanding of the molecular mechanisms of VV embryological development, maintenance after formation, and failure in disease is limited, and there are few therapeutic options to treat VV dysfunction (3, 11–16). Elucidating these mechanisms and understanding how their dysfunction may lead to VV failure could facilitate the development of novel therapies.

Clinical studies have suggested a link between venous reflux and some primary lymphedemas, and we have previously shown striking human VV disease in patients with primary lymphedema caused by mutations in *FOXC2* (MIM 602402) and *GJC2* (MIM 608803; refs. 11, 17–20). Other human genetics studies have shown that mutations in the gene encoding the tyrosine kinase receptor EPHB4 (*EPHB4,* MIM 618196) cause capillary malformation–arteriovenous malformation syndrome (CM-AVM2, including hereditary hemorrhagic telangiectasia and vein of Galen malformations, cutaneous malformations and arteriovenous malformations) and a primary lymphatic anomaly, which includes clinical features such as central conduction lymphatic anomaly, nonimmune fetal hydrops, and atrial septal defects (21–28). Patients with the primary lymphatic anomaly were also reported to present with varicose veins and early onset venous stasis (21, 25, 28). In mice, early embryonic deletion of *Ephb4* in lymphatic endothelia leads to subcutaneous edema and abnormal dermal and mesenteric lymphatic vasculature, whereas deletion in adult blood endothelia results in coronary abnormalities including capillary microhemorrhages (21, 29)

The Eph receptors are the largest family of mammalian receptor tyrosine kinases and bind to ephrins, their transcellular ligands (30, 31). Cell-cell signaling may occur in either direction, resulting in cell and context-specific effects, and is involved in regulating many developmental processes including cell sorting and boundary formation (32–34). In the cardiovascular system, ephrinB2 is widely accepted as an arterial-specific marker, whereas EphB4 is used as a marker of venous endothelia (35–37). EphrinB2/EphB4 signaling is essential for developmental angiogenesis, and global knockout of *Ephb4* is phenotypically similar to knockout of *Efnb2*, with both resulting in vascular remodeling defects and embryonic lethality (35, 38–40). Constitutive overexpression of ephrinB2 leads to defects including abnormal intersomitic vessel patterning, aortic dissection and aneurysm formation, and early neonatal lethality due to aortic rupture (41)

Signaling between ephrinB2 and EphB4 is required for lymphatic valve (LV) development and maintenance, and for formation of valves at lymphovenous junctions at the base of the neck (12, 21, 42). LV cells fail to take on normal morphology in *Efnb2*^*ΔV/*^*ΔV* mice (lacking the C-terminal PDZ interaction site), and it was suggested that ephrinB2/EphB4 signaling is required to guide endothelial cell (EC) migration and elongation during LV morphogenesis (42). Blocking the forward signaling activity of EphB4 results in failure of LV formation (43, 44). Defects in cardiac valve (CV) development leading to early perinatal death are found in *Efnb2*^*βgal/*^*βgal* mice, in which the cytoplasmic tail of ephrinB2 is replaced with βgal (45). In both LV and CV, the morphological effects of loss (or inhibition) of ephrinB2/EphB4 signaling on Prox1^hi^ valve-forming cells (VFCs) remain unclear. Ephrin–Eph interactions result in rapid changes in cellular direction and motility, leading to boundary formation within initially mixed populations of cells (for example, in mesenchymal cells), and can inhibit communication via gap junctions across these boundaries (31, 32, 46). In vitro, ephrinB2/EphB4 signaling controls EC repulsion and segregation, leading to clustering of EphB4-expressing or ephrinB2-expressing cells, akin to in vivo boundary formation, but to the best of our knowledge this behavior has not been observed in ECs in vivo (47)

We previously showed that ephrinB2 is required for postnatal VV leaflet development and maintenance, but the expression of ephrinB2 and EphB4, and any roles in the early organization of VFCs, has not been examined (11, 12). In this study, we show that mutations in *EPHB4* caused striking human VV disease, with an almost complete loss of VVs seen in some patients. Given the known roles for ephrin–Eph interactions in boundary formation in other tissues, we hypothesized that ephrin–Eph interactions could regulate early organizational events in VV formation. We have therefore focused on their respective roles in the regulation of the complex series of events during early valve formation in mice, which includes the organization of a set of Prox1^hi^ VFCs to form a ring of cells within the 3D lumen of the vessel (stage 1 of development; refs. 11, 12). On postnatal day 0 (P0) this structure is found predominantly on the anterior vein wall and then extends posteriorly (11). Using a GFP reporter we identified *Efnb2* expression within veins at the site of VV formation, and that the organization of VFCs occurred at a striking boundary between venous ECs that expressed ephrinB2 and those that did not. A conditional loss-of-function genetic approach has enabled us to show that both ephrinB2 and EphB4 were required for these early organizational events and that EphB4 was required for postnatal VV development.

## Results

### Patients with mutations in EPHB4 had fewer VVs and showed deep venous reflux.

**Figure 1:**
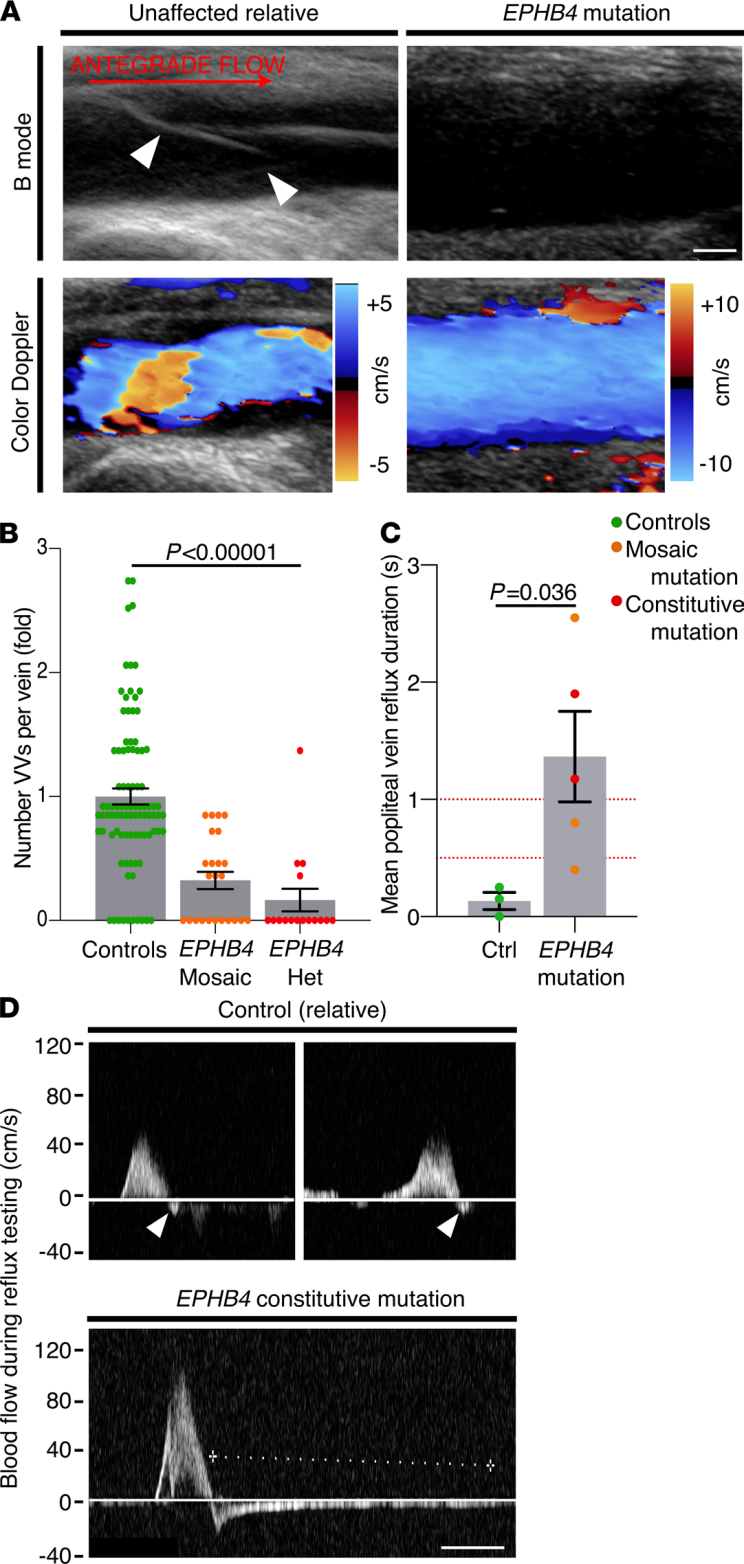
EPHB4 mutations cause human VV failure. (**A**) VVs (arrowheads) were readily identifiable in the veins of controls, including an unaffected relative, but were rare in patients with a mutation in *EPHB4*. B-mode and color Doppler images are shown of the popliteal vein. Blood flow left to right, velocity indicated by color scale. Scale bar: 2 mM. (**B**) Fewer VVs per vein were seen in participants with mosaic or constitutive (heterozygous) *EPHB4* mutation (*P* = 1.7 × 10^–11^, 1-way ANOVA). *n* = 92 veins in 13 controls, and 40 veins in 5 patients with *EPHB4* mutation (mosaic or constitutive). Data points represent individual veins. (**C**) Popliteal (deep) venous reflux was identified in mosaic and constitutive carriers of *EPHB4* mutations (*P* = 0.036, Mann-Whitney *U* test). Blood velocity ≥ 0.5 second indicates reflux and ≥ 1 seconds indicates severe reflux (red dotted lines). Data points represent mean of left and right popliteal reflux duration for each individual. (**D**) Representative blood velocity in the popliteal vein during reflux testing is shown for an unaffected relative (with no significant reflux, arrowheads) and a patient carrying an *EPHB4* mutation, demonstrating significant deep venous reflux (dotted line = 2.14 seconds). Scale bar: 500 ms). Throughout all figures, antegrade blood flow is from left to right. Data are shown as mean ± SEM. VVs, venous valves; Het, heterozygous.****

Pathogenic mutations in *EPHB4* were recently described in 2 families with primary lymphatic-related fetal hydrops (LRFH), with autosomal dominant inheritance (21). Adults in both families had a notably early onset of lower limb venous disease. We therefore characterized the numbers of valves per vein in these patients (*n* = 5) and an unaffected relative using ultrasonography, and compared these results with a control population (*n* = 12; Supplemental Table 1; supplemental material available online with this article; https://doi.org/10.1172/jci.insight.140952DS1). VVs were readily detected in the unaffected relative and other controls, but fewer VVs were detected in patients carrying a heterozygous mutation in *EPHB4,* including 3 patients with a mosaic mutation in *EPHB4* (fold change 0.2 ± SD 0.29 for mosaic carriers, and 0.17 ± 0.36 for constitutive carriers, *P* = 1.7 × 10^–11^, 1-way ANOVA, *F* = 30.3, 2 df; Figure 1, A and B, and Supplemental Figure 1). Ninety-two veins were analyzed in 13 controls, and 40 veins were analyzed in 5 mosaic or constitutive *EPHB4* mutation carriers. Given the substantial loss of VVs in those with constitutive *EPHB4* mutations, too few VVs were available for detailed analysis of leaflet length in constitutive mutation carriers, but those VVs that were identified were not significantly shorter than controls (Supplemental Figure 1; fold change 1.15 ± 0.63 for mosaic carriers, and 0.67 ± 0.48 for constitutive carriers, *P* = NS). Groups were matched for age and sex (*P* = NS). Those carrying an *EPHB4* mutation had a mean popliteal reflux duration of 1.37 seconds, above the accepted diagnostic threshold of 1 second for severe deep venous reflux. Both patients with constitutive *EPHB4* mutations exhibited a mean popliteal vein reflux duration of 1 second or longer (Figure 1, C and D, and Supplemental Figure 1).

### EphB4 was expressed on E18 and P0 and was required for normal VFC organization.

EphB4 is the main ephrinB2 receptor in the vasculature, and these proteins often exhibit a complementary expression pattern during tissue segmentation (13, 35). Our analysis initially focused on embryonic day 18 (E18) and P0. We localized EphB4 expression in the region of the developing valve in *Efnb2^GFP^* mice and then examined whether EphB4 was required for organization of VFCs on P0.

**Figure 2:**
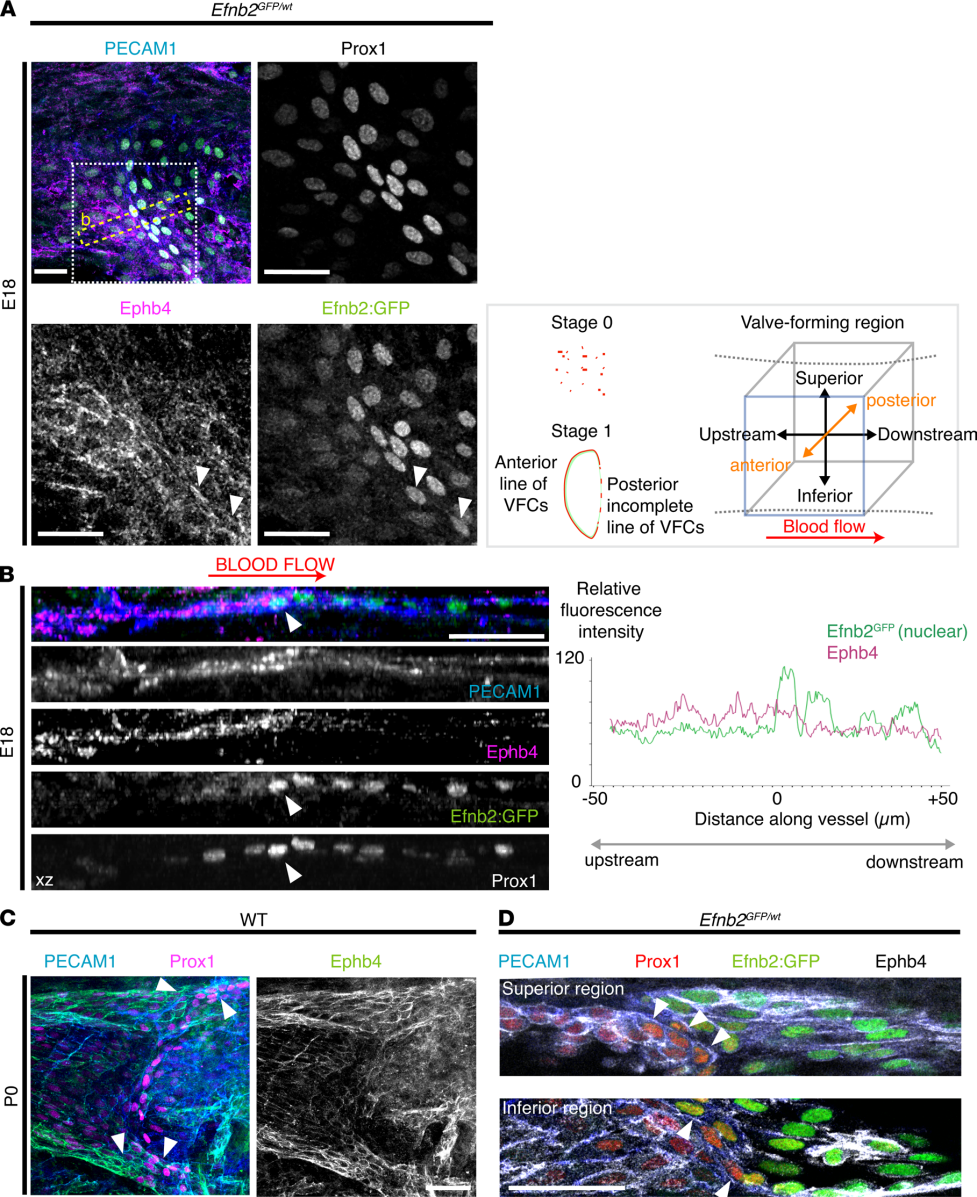
Expression of EphB4 in Efnb2GFP reporter E18 and P0. (**A**) Localization of PECAM1 (blue), Ephb4 (magenta), Prox1 (white), and Efnb2:GFP (green) in heterozygous Efnb2^GFP^ mice on E18. Part of an E18 VV is shown, and the white boxed area (which contains organizing VFCs) is shown enlarged in single channel images. Only the anterior vein wall is shown. Arrowhead indicates a VFC nearer the inferior edge of the vessel coexpressing Ephb4 and Efnb2GFP. The schematic indicates stages 0 and 1 of VV development, as previously defined in ref. 11. Red = Prox1^hi^ VFCs, which form a continuous line across the anterior vein wall at stage 1. The orientation of all confocal *Z* stacks is indicated and is the same throughout all figures. (**B**) An XZ projection (13.6-μm deep) and the fluorescence intensity profile for Efnb2GFP and EphB4 are shown across the organizing VFCs, indicated by the yellow boxed area in **A**. The EphB4 signal is stronger upstream (to the left) of the VFCs (indicated by arrowheads, or “0” on the graph x axis), whereas the Efnb2GFP signal is stronger in VFCs and downstream (*P* < 0.0001, *n* = 6 VVs, 2-tailed *t* test). The multichannel image does not include Prox1. (**C**) In WT VVs on P0, Prox1^hi^ VFCs expressed EphB4, and it was particularly strongly expressed in the superior and inferior areas of the vein (arrowheads). (**D**) Coexpression of Ephb4 and Efnb2 was confirmed in *Efnb2GFP* mice. *Z* projections (6 μm) of the upper and lower regions of a valve are shown. Arrowheads indicate reorientated VFCs (orange). (Uncropped images are provided in Supplemental Figure 2B.) Scale bars: 20 μm. VFCs, valve-forming cells; VVs, venous valves; E18, embryonic day 18; P0, postnatal day 0.

On E18, when VFCs were in the process of organizing themselves at the site of developing valves, EphB4 expression appeared to be stronger immediately upstream of areas showing VFC organization and adjacent to VFCs with high *Efnb2* expression (Figure 2A and Supplemental Figure 2C). Quantification of *Efnb2^GFP^* signal and EphB4 immunosignal across these organizing areas (yellow box in Figure 2A) confirmed relatively complementary expression with significantly higher EphB4 upstream and higher *Efnb2^GFP^* downstream of the VFCs (Figure 2B). Conversely, VFCs nearer the superior or inferior edges of the vessel already coexpressed *Efnb2^GFP^* and EphB4 (arrowheads in Figure 2A).

By P0, VFCs consistently reorientated and elongated to form a line of cells across the anterior femoral vein wall and partly extended across the posterior wall, defined as stage 1 of VV development (schematic in Figure 2A). Prior to this, development is described as stage 0. We had thought that EphB4 expression would be complementary to *Efnb2* expression on P0, but EphB4 was immunolocalized variably throughout the valve region, with stronger expression within clusters of VFCs at the superior and inferior regions of the valve (Figure 2C, arrowheads), where we previously identified multiple proliferating VFCs (11). Coexpression of Ephb4 and *Efnb2* was confirmed in *Efnb2^GFP^* mice (Figure 2D and Supplemental Figure 2B).

**Figure 3:**
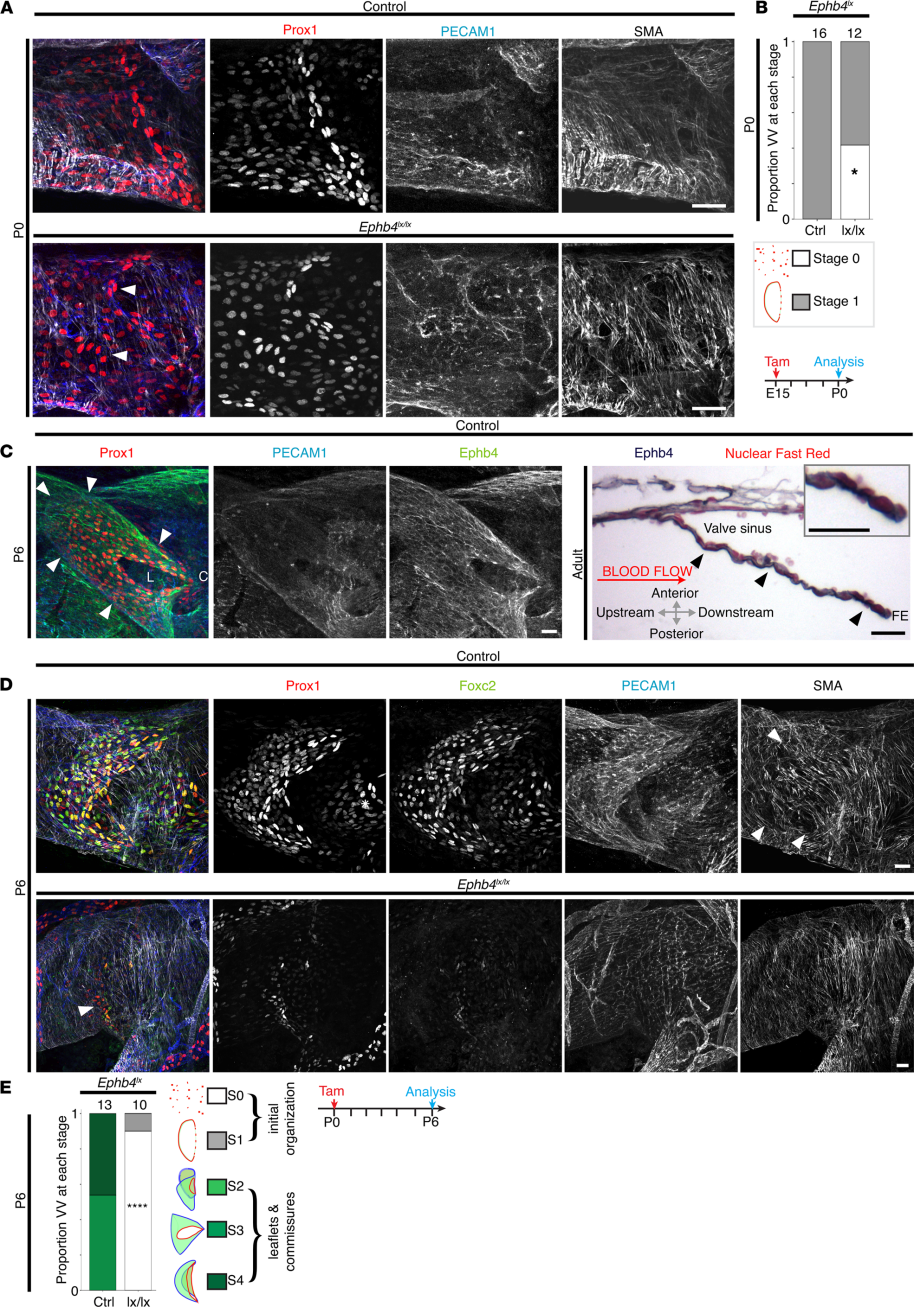
EphB4 is expressed on E18 and P0 and is required for normal VFC organization and leaflet development to P6. (**A** and **B**) Homozygous deletion of *Ephb4* on E15 (analyzed on P0) resulted in disrupted organization of VFCs, similar to deletion of *Efnb2*, albeit some VVs appeared to develop normally. The number of VVs analyzed for each condition is indicated above each bar in the chart. *P = 0.008, Fisher’s exact test. Scale bars: 20 μm. (**C**) EphB4 was localized in WT P6 VVs and surrounding vein. The leaflet of a stage 3 VV is indicated by arrowheads. L = valve lumen and C = the single commissure. In adult VVs, longitudinal sections were prepared, and EphB4 (dark blue stain) was most strongly localized to the luminal surface of VV leaflets (black arrowheads) and leaflet free edge (enlarged in inset). The counterstain is Nuclear Fast Red. Arrows indicate the orientation of the adult histological section only (all confocal images are oriented as shown in Figure 2). (**D** and **E**) Induction of homozygous *Ephb4* deletion on P0 with tamoxifen (analysis on P6) resulted in entirely absent VV leaflets and failure to remodel the surrounding SMCs (arrowheads in upper panel) on P6. Only a few Prox1^hi^/Foxc2^hi^ cells remained (arrowhead in lower panel). The asterisk indicates a downstream tributary valve. (**E**) Bar chart shows the proportion of VVs identified at each stage, with stage and color indicated in adjacent key, on P6 for the indicated genotypes. The number of VVs analyzed for each condition is given above each bar. ****P < 0.00005, χ^2^ vs. control, *n* = 13 control VVs vs. 10 *Ephb4* deleted. Scale bars in **A**, **C**, and **D**: 20 μm. VFCs, valve-forming cells; VVs, venous valves; E18, embryonic day 18; P0, postnatal day 0; Tam; tamoxifen; SMA, smooth muscle α-actin; SMC, smooth muscle cell.

Deletion of *Ephb4* on E15 resulted in disorganized VFCs on P0 (Figure 3A) albeit some VVs developed normally to stage 1 (Figure 3B).

### Ephb4 was required for leaflet development to P6.

We next localized the expression of EphB4 in VV leaflets on P6 and in adult mice. We then examined whether EphB4 is required for maturation of the valve leaflets up to P6.

EphB4 continued to be expressed in the endothelia of veins and VV leaflets on P6 and in adults (Figure 3C, left panel). Expression was strongest on the lumen surface of the valve leaflet, including cells at the free edge of valve leaflet (Figure 3C, right panel). This expression is complementary to the previously identified lack of expression of *Efnb2* in these free-edge cells (12). This could contribute to maintenance of their phenotype, which is clearly different to the rounded morphology of endothelia lining the sinus or lumen leaflet surfaces (12)

On P6 VVs are normally at stages 3 or 4 (schematic in Figure 3), which were defined, as previously, by the presence of 1 or 2 commissures (11). Deletion of *Ephb4* on P0 led to a complete failure of valve leaflet formation by P6, with only a few Prox1-expressing or Foxc2-expressing cells remaining (Figure 3, D and E). This phenotype (*Ephb4* deletion on P0, analyzed on P6) was more consistent and severe than deletion on E15, analyzed on P0 (Figure 3A).

Similar to other gene-deletion studies resulting in loss of VFCs by P6, there was an associated failure to establish a local reduction in the density of smooth muscle cells (SMCs) around the valve (Figure 3D; ref. 11)

### VFC organization occurred at a developing boundary between ECs expressing and not expressing Efnb2.

**Figure 4:**
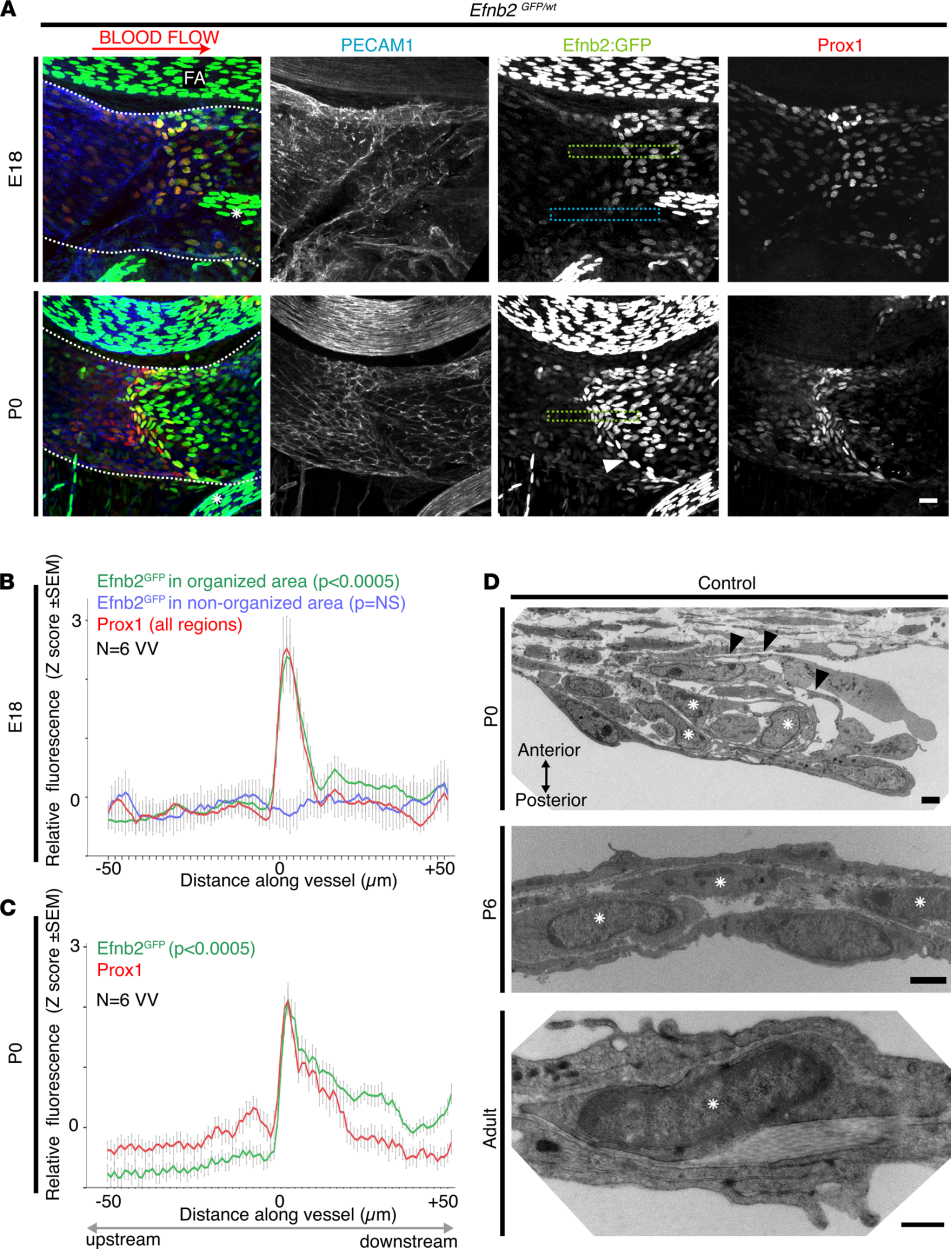
Formation of ephrinB2 expression boundary in VV-forming region. (**A**) Localization of PECAM1 (blue), Prox1 (red), and Efnb2GFP reporter signal (green, His-tagged and therefore nuclear) on E18 and P0 in heterozygous Efnb2GFP mice. Wholemount preparation of the proximal femoral vein is shown. On E18 there was partial, and variable, organization of VFCs, for example, in the superior area of the VV-forming region but not the inferior area. Those areas with organization on E18 showed a weak Efnb2GFP expression boundary, which was clearer on P0 (white arrowhead). Dotted lines indicate the femoral vein boundary, adjacent to the femoral artery. As expected, arterial endothelial cells showed stronger Efnb2GFP signal. *Indicates an overlying arterial branch (cut). (**B** and **C**) On E18, analysis of the relative fluorescence intensity across developing valves revealed a peak in Efnb2GFP signal (green line) coincident with that of Prox1^hi^ (red) VFCs in organizing areas, but not in adjacent areas that are not yet organized (blue line). At both E18 and P0, Efnb2GFP signal is stronger downstream, and this difference is more apparent on P0. Mean of 6 VVs and 7–12 regions analyzed per VV and representative regions analyzed are shown boxed (green, blue) in **A**. *P*s in **B** and **C** are 2-tailed *t* tests comparing Efnb2GFP proximal and distal to the VFC leading edge. NS. (**D**) TEM analysis on P0 showed rotated VFCs detached from underlying basement membrane (arrowheads). Interstitial cells (*) populated the developing leaflet core, and persisted on P6 and in adults. TEM micrographs are orientated at 90°C to confocal images, as indicated by arrows on P0 in **D**. Further examples of interstitial cells (in murine and human VVs) are shown in Supplemental Figure 3. *n* ≥ 6 VV and blood flow left to right at all time points and in **B** and **C**. Scale bar in **A** is 20 μm and scale bar in **D** is 2 μm on P0–P6, 500 nm in adults. VFCs, valve-forming cells; VVs, venous valves; E18, embryonic day 18; P0, postnatal day 0; TEM, transmission electron microscopy.

To visualize the *Efnb2* expression pattern during VFC organization, we visualized the site of VV formation in the proximal femoral vein using confocal microscopy of wholemount samples from *Efnb2^GFP^* reporter mice (Figure 4A). *Efnb2GF*^P^ signal was strong in femoral artery ECs (Figure 4A), and generally absent or at very low levels in venous endothelia in all samples analyzed, similar to previously reported findings (36, 37). Expression of *Efnb2* by venous smooth muscle α-actin–expressing mural cells was not detected (data not shown). Global heterozygous knockout of *Efnb2* (in the *Efnb2^GFP^* reporter) did not prevent development of stage 1 VVs by P0 (*P* = NS versus WT littermates, *n* = 32 *Efnb2^GFP/wt^* VVs analyzed). On E18 the patterning of Prox1^hi^ VFCs within the valve-forming region was more variable than on P0, with areas of Prox1^hi^ cells (e.g., the superior but not inferior area) showing organization (i.e., reorientation and elongation of cells; Figure 4A, upper panel versus lower panel). The organizing VFCs, and endothelia just downstream of organizing VFCs, expressed *Efnb2* (Figure 4A, green box), whereas areas without VFC organization did not develop a boundary in ephrinB2 expression (Figure 4B, blue box). Quantification of the *Efnb2^GFP^* signal on E18 confirmed a boundary in expression of *Efnb2* in regions of organized cells, but not in adjacent nonorganized regions (Figure 4B).

On P0 *Efnb2* was consistently expressed (and more strongly than on E18) by the line of Prox1^hi^ VFCs and in cells downstream, but not upstream, of the VFCs (Figure 4A, lower panel). Quantification confirmed the boundary in *Efnb2^GFP^* signal, with a peak in *Efnb2* expression coinciding with Prox1^hi^ VFCs (Figure 4C). Whereas on E18 the downstream *Efnb2^GFP^* signal was marginally higher than the upstream signal (Figure 4, A and B), on P0 this difference was more marked (Figure 4, A and C). These results suggest that the *Efnb2* expression boundary was formed concomitantly with the organization of Prox1^hi^ VFCs and suggest that an Eph–ephrin interaction within venous endothelia might have participated in the regulation of VFC organization.

Analysis of this valve-forming region on P0 in WT mice by transmission electron microscopy (TEM) demonstrated that development of the core of the valve leaflet is more advanced than previously characterized, with the presence of interstitial cells within the leaflet, which is already protruding from the vessel wall (Figure 4D, upper panel; ref. 11). VFCs at the leading edge of the protruding leaflet were partly detached from the underlying basement membrane, consistent with their progressive reorientation and migration (Figure 4D, upper panel, arrowheads), which has been previously identified in developing LV (48). TEM analysis on P6 and in adult mice confirmed the presence of interstitial cells in murine VV (Figure 4D, middle and lower panels, and Supplemental Figure 3, A–C), consistent with their known presence in, for example, rabbit VV (2). The presence of interstitial cells in human VV was confirmed by TEM and histology (Supplemental Figure 3, D and E). Connexin43 (Cx43) and Connexin47 (Cx47), proteins implicated in human VV disease (11), were immunolocalized to human VV interstitial cells (Supplemental Figure 3F).

### Efnb2 was required for normal VFC organization.

**Figure 5:**
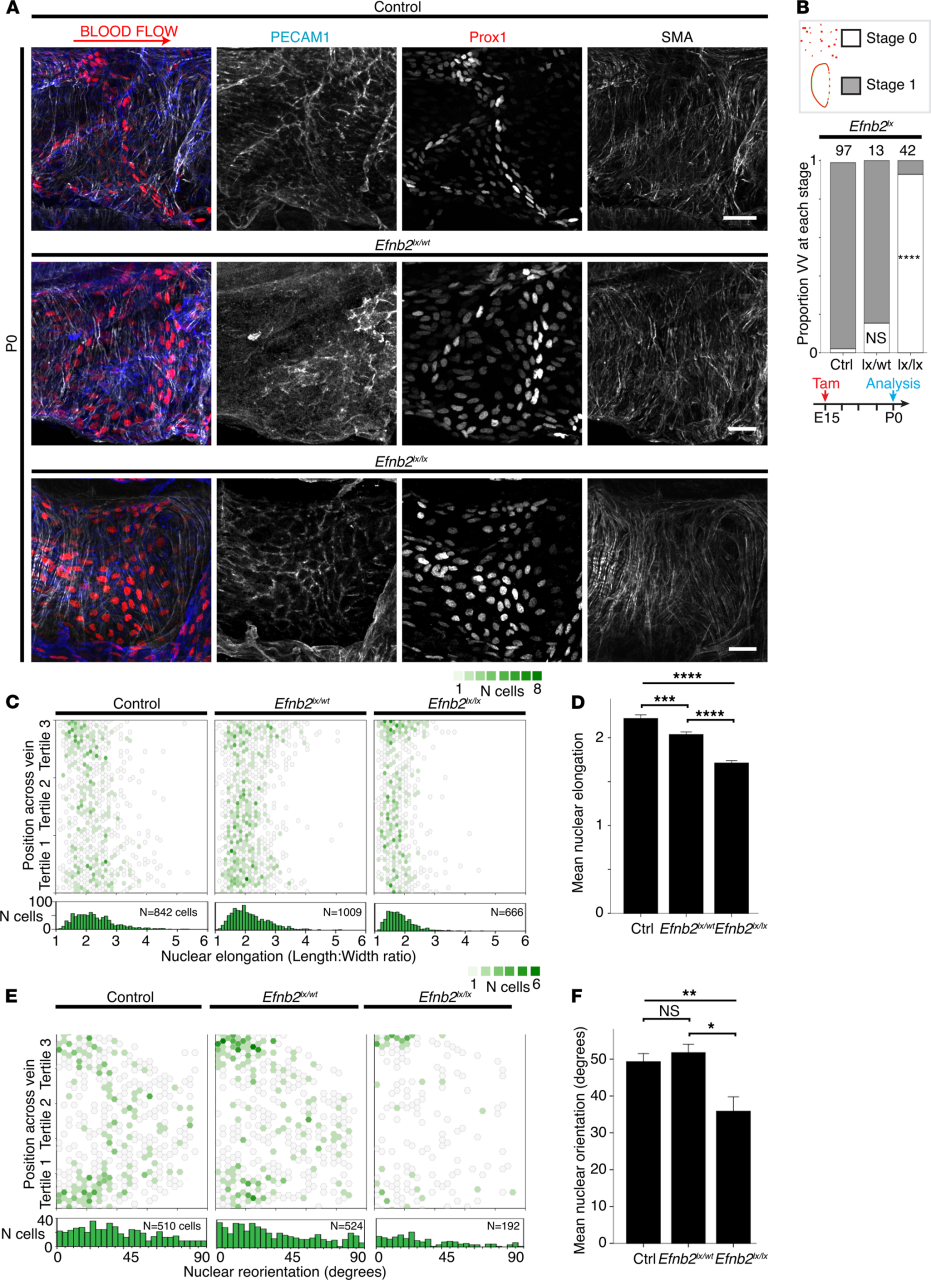
Effect of Efnb2 deletion on organization of VFCs. (**A** and **B**) Localization of PECAM1 (blue), Prox1 (red), and SMA (white) in littermate controls and heterozygous (*Efnb2^lx/wt^*) and homozygous (*Efnb2lx/lx*) mice on P0, after tamoxifen induction of Efnb2 deletion on E15. In controls and *Efnb2^lx/wt^* mice, valves reached stage 1 of development, as normal. Homozygous deletion resulted in a failure to organize normally, with Prox1^hi^ cells distributed over a wider upstream–downstream area of the vein and failure of VFCs to elongate and reorientate. (**B**) The bar chart shows the proportion of VVs identified at stage 0 (white) and stage 1 (grey) on P0 for the indicated genotypes, and the number of VVs analyzed for each condition is given above each bar. *P*s derive from 2-sided Fisher’s exact test vs. control. (**C**) Hex-binned scatterplot of VFC elongation (length/width ratio) across the vein from superior to inferior. *n* = 2517 cells, ≥ 6 VVs. (**D**) Bar chart (± SEM) summarizing the results from **C** showing that both heterozygous and homozygous deletion resulted in significant reductions in VFC elongation. One-way ANOVA with Bonferroni’s post hoc test. For between groups 1-way ANOVA, F = 109 with 2 df, P = 3.2 × 10^–46^. (**E**) Hex-binned scatterplot of VFC reorientation (in VFCs with nuclear length/width ratio ≥ 2) across the vein from superior to inferior. *n* = 1226 cells, ≥ 6 VVs. After homozygous deletion, the VFCs with correctly reorientated nuclei were lost, particularly in the center of the vessel. (**F**) Bar chart (± SEM) summarizing the results from **E**. Homozygous deletion resulted in significantly reduced reorientation. One-way ANOVA with Bonferroni’s post hoc. For 1-way ANOVA, *F* = 7.1 with 2 df, *P* = 0.0009. *P < 0.05, **P < 0.005, ***P < 0.0005, ****P < 0.00005. Scale bars: 20 μm. VFCs, valve-forming cells; VVs, venous valves. SMA, smooth muscle α-actin.

Having established the expression pattern of *Efnb2* during VFC organization, we then examined whether *Efnb2* is required for the organization of VFCs on P0. We performed conditional gene deletion using floxed *Efnb2* alleles and *Prox1Cre^ERT2^,* and quantified each valve according to developmental stage and also quantified the elongation and reorientation of Prox1^hi^ cells (as previously described in refs. 11, 12, 48). Heterozygous deletion on E15 did not significantly affect VV development to stage 1 (Figure 5A, middle panel, and Figure 5B). There was, however, a small but significant reduction in VFC nuclear elongation (Figure 5, C and D), but no difference in their reorientation (Figure 5, E and F). Homozygous deletion of *Efnb2* resulted in disorganized VFCs that failed to reach stage 1 of development on P0, with a similar pattern of disorganization to that seen with deletion of *Ephb4* (*P* < 0.001; Figure 5A, lower panel, and Figure 5B). Prox1^hi^ cells were present but appeared to be distributed across a wider upstream–downstream region of the vessel, and exhibited markedly reduced elongation (Figure 5, C and D; *P* < 0.00005) and reorientation (*P* < 0.005; Figure 5, E and F). These findings demonstrate that endothelial *Efnb2* was required for the normal organized patterning of VFCs on P0.

These results, together with those we described for *Ephb4*, show that the expression of EphB4 and *Efnb2* was dynamic during VV organization, and complementary expression (*Efnb2* higher downstream, EphB4 higher upstream) occurred during the process of organization on E18, but by P0, VFCs expressed both *Efnb2* and EphB4.

### Efnb2 was required for projection of VFCs into the vessel lumen, normal expression of integrin α9, and normal polarity.

**Figure 6:**
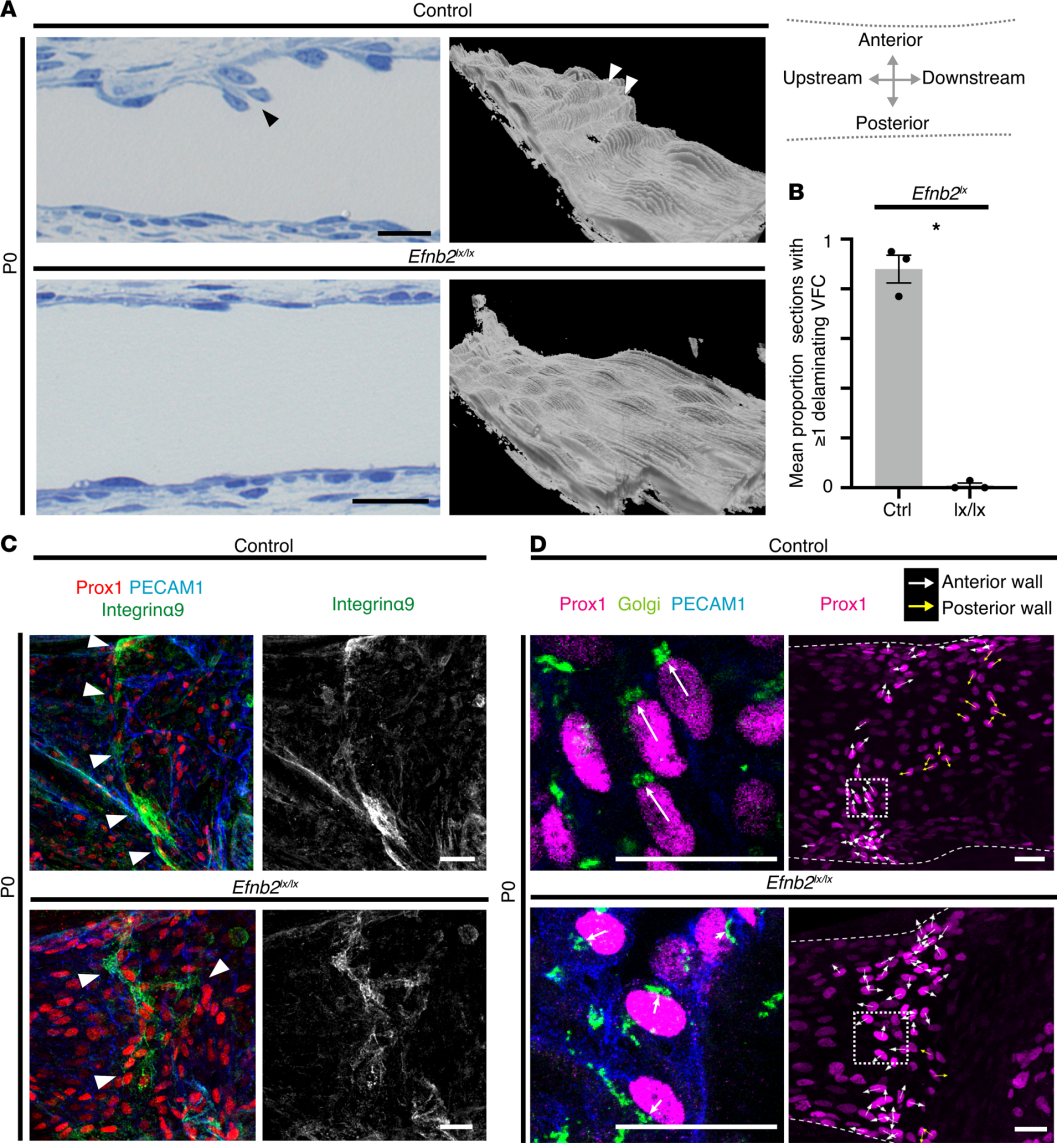
Failure of VFCs to project into vessel lumen and abnormal integrin expression. (**A**) Semithin longitudinal sections of P0 femoral veins showed protruding VFCs in littermate controls, but no protruding cells were seen after homozygous *Efnb2* deletion. 3D reconstructions of semithin sections show protruding VFCs (arrowheads) in controls only. The schematic indicates the orientation of the semithin sections. (**B**) A significant reduction in the mean number of sections showing protruding cells was identified (≥ 60 sections were analyzed per sample, *P < 0.05 by 2-tailed *t* test, *n* = 3 VV per group, data are shown as mean ± SEM). (**C**) Integrin α9 was expressed in a ring around the organized VFCs in littermate controls (white arrowheads), but after homozygous *Efnb2* deletion, the localization of integrin α9 expression was disrupted and chaotic (*P* < 0.05, χ^2^ test of the proportion of VVs showing normal vs. disrupted integrin α9 expression pattern, *n* ≥ 6 VV per group). (**D**) VFC polarity (indicated by white arrows) was examined by costaining for Prox1 (magenta), PECAM1 (blue), and Golgi (green). Polarity was determined for individual VFCs using 0.5-μm sections, and a *Z* projection of 2–4 confocal sections shown on the right (area enlarged outlined by dotted box,). In littermate controls, cells in the central region of the vein were aligned with the line of organized VFCs, whereas after homozygous *Efnb2* deletion, cell alignment was chaotic. *P* < 0.05, χ^2^ test of the proportion of VVs showing normal vs. chaotic VFC alignment, *n* ≥ 8 VV per group. Yellow arrows indicate VFCs on the posterior vein wall. Scale bars in **A**, **C**, and **D**: 20 μm. **C** and **D** are oriented as shown in Figure 2A. VFCs, valve-forming cells; VVs, venous valves.

We prepared longitudinal semithin sections in the XZ-plane of the wholemount preparations, to more clearly examine projection of VFCs into the vessel lumen. Compared with littermate controls, VFCs failed to project into the vessel lumen in homozygous *Efnb2*-deleted cells (Figure 6, A and B). We hypothesized that failure to correctly express integrin α9 could be a mechanism underlying the failure of VFCs to organize and project into the lumen in *Efnb2*-deleted mice, because integrin α9 is required in valve formation for extracellular matrix remodeling, and for VV leaflet growth and maintenance (11, 12, 49). On P0 integrin α9 expression was largely localized to the line of VFCs on the anterior vein wall (Figure 6C, upper panel). After *Efnb2* deletion, the integrin α9 expression pattern followed the abnormal, broader distribution of the Prox1^hi^ cells, and appeared haphazard (Figure 6C), likely precluding normal matrix remodeling.

Because VFCs appeared in a broader region after *Efnb2* deletion, we hypothesized that without guidance from ephrin–Eph interactions on E18, these cells would be disorientated on P0. In LV formation, lymphatic ECs elongate and migrate centrally from the edges of the vessel (48, 50) and in migratory ECs, the Golgi apparatus is positioned apically of the nucleus (51). We therefore analyzed VFC alignment by costaining for a Golgi marker, and examined the alignment of cells with the forming VV structure (Figure 6D, upper panel). In littermate controls on P0, cells were consistently aligned across the vessel anterior wall, whereas in all samples with homozygous *Efnb2* deletion, there was a disrupted pattern (Figure 6D, lower panel).

### Gap junction intercellular communication and proliferation.

**Figure 7:**
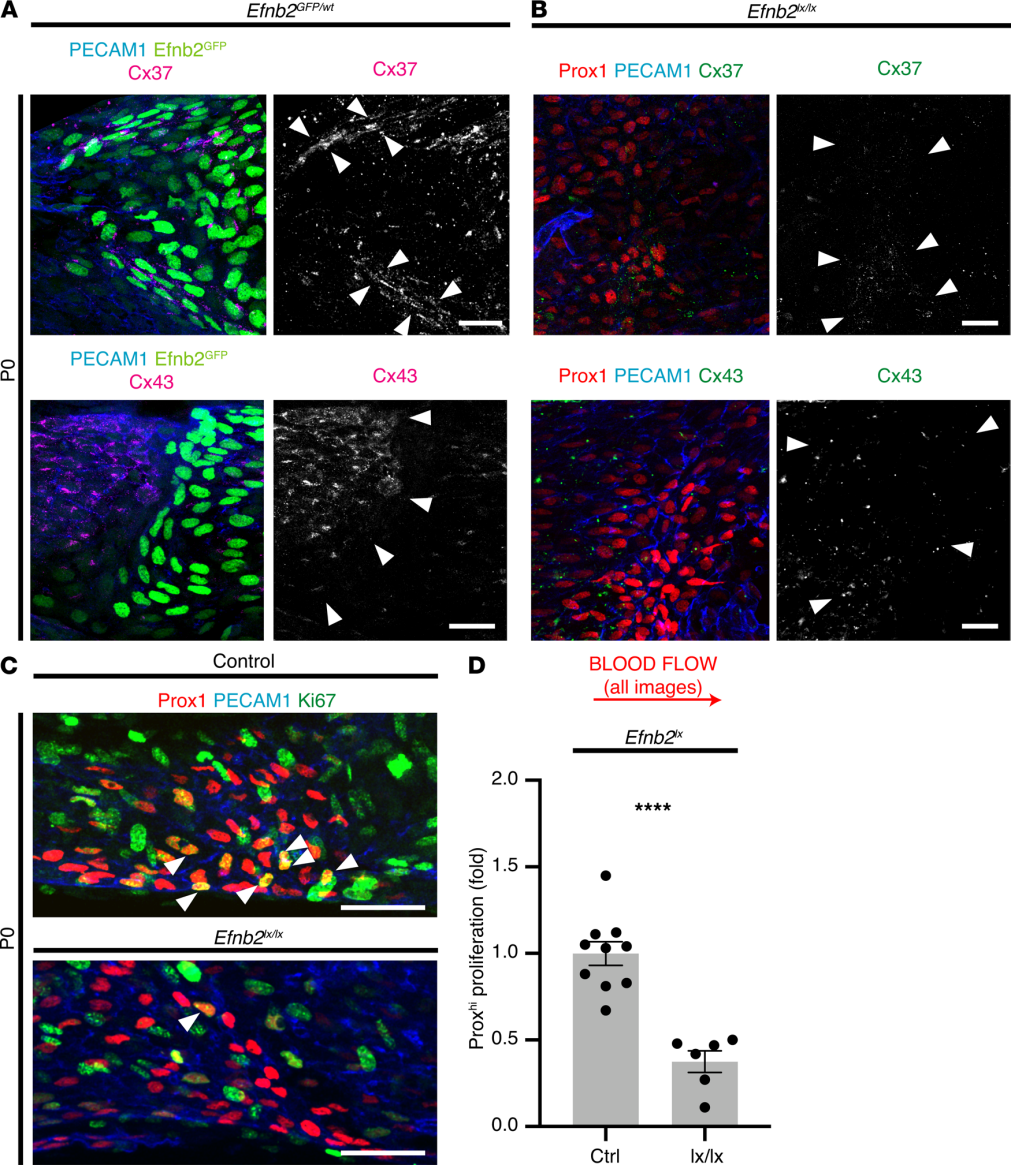
*Efnb2* deletion disrupts gap junction protein expression pattern and proliferation. (**A**) Localization of PECAM1 (blue), and either Cx37 or Cx43, as indicated (magenta) around VFCs on P0 in heterozygous Efnb2GFP (green) mice. As expected, on P0 Cx37 was localized to Efnb2GFP-expressing VFCs, primarily forming large gap junction plaques (examples indicated between arrowheads) and Cx43 was localized to endothelium upstream of these VFCs (region to the left of the arrowheads). Smaller plaques are also identifiable. (**B**) Localization of PECAM1 (blue), Prox1 (red), and either Cx37 or Cx43, as indicated (green), after homozygous deletion of *Efnb2*. The tightly regulated expression pattern of Cx37 was disrupted, with expression over a wider area (arrowheads) and the typical appearance of larger plaques was lost. The expression pattern of Cx43 was also disrupted and no longer confined to upstream of VFCs (arrowheads; *P* < 0.05, χ^2^ test of the proportion of VVs showing normal [confined] vs. disrupted expression pattern, *n* ≥ 6 VV per group). (**C** and **D**) The proportion of proliferating VFCs was assessed by colocalization of Prox1 and Ki67 (arrowheads). Ki67^+^ VFCs were easily identified in littermate controls, but far fewer proliferating VFCs were identifiable after homozygous *Efnb2* deletion. The inferior region of the vein is shown; ****P < 0.00005, unpaired 2-tailed *t* test, *n* ≥ 6 VV per group, data are shown as mean ± SEM). Scale bars in **A–C**: 20 μm. VFCs, valve-forming cells; VVs, venous valves; P0, postnatal day 0.

Expression of ephrinBs may regulate cell behavior by modulating connexin communication domains, including via Cx43 (32, 46). Cx43 and Connexin37 (Cx37) have highly regulated expression patterns around VFCs on P0, and both are required for venous, lymphatic, and lymphovenous valve formation (11, 13, 14, 52–54). Large gap junction plaques containing Cx37 are normally expressed by Prox1^hi^ VFCs on P0, whereas Cx43 is primarily expressed in a region just upstream of the organized VFCs on P0. Homozygous deletion of Cx43 (using *Prox1Cre^ERT2^*), or homozygous knockout of Cx37, results in a failure of organization of VFCs on P0, which is reminiscent of the phenotype seen with homozygous deletion of *Efnb2* (11, 13, 14). This failure of organization on P0 is followed by complete loss of valve structure (13). We therefore examined the expression patterns of Cx37 and Cx43 relative to *Efnb2* expression in the *Efnb2^GFP^* reporter mice and after homozygous deletion of *Efnb2*. The normally highly restricted expression patterns of Cx37 and Cx43 were disrupted on P0 after homozygous *Efnb2* deletion (Figure 7, A and B). In the *Efnb2^GFP^* reporter, Cx37 localization indicated gap junction plaque formation around *Efnb2*-expressing VFCs (Figure 7A, white arrowheads in upper panel), whereas after *Efnb2* deletion, no plaque formation was identified (or possibly plaques were very much smaller), and Cx37 expression appeared more widespread in the region of the VFCs (Figure 7B).

In previous genetic loss-of-function experiments (including knockout of Cx37), disruption of VFC organization was associated with a reduction in VFC proliferation (11), and so we next examined whether deletion of *Efnb2* altered VFC proliferation or apoptosis. As previously described, Ki67^+^-proliferating VFCs appeared more abundant in the superior and inferior regions of the valve on P0 (11). A reduction in the proportion of proliferating VFCs was seen after *Efnb2* deletion (*P* < 0.001; Figure 7, C and D), but no effect on apoptosis (as detected by Caspase-3 expression) was observed (data not shown).

## Discussion

We have identified human VV failure and deep venous reflux caused by mutations in *EPHB4*. This phenotype was more severe (i.e., a greater loss of valves) than that previously identified in patients with mutations in *FOXC2* or *GJC2* (a fold change versus controls of 0.2 ± SD 0.29, mosaic *EPHB4*, or 0.17 ± 0.36, constitutive heterozygous *EPHB4*, for the reduction in mean VVs per vein; ref. 11). Almost all of these patients did not have clinical evidence of chronic lower limb primary lymphedema. Some presented with nonimmune fetal hydrops, which was of lymphatic origin, but it had resolved soon after birth. After that, their most obvious clinical sign of disease was early onset prominent or varicose veins, and venous insufficiency (21). We now know that this is venous valvular aplasia, and therefore mutations in *EPHB4* should be considered as a cause of primary venous valvular aplasia (5–8, 21, 55). Dysfunction of the deep VVs increases the rate of progression of chronic venous insufficiency, with a higher rate of chronic venous ulcer formation. The management of deep venous reflux is extremely challenging, because currently there are no reliably effective therapies beyond invasive surgical construction of neovalves (3)

Heterozygous mutations in *EPHB4* are reported to cause CM-AVM2, vein of Galen aneurysmal malformation, LRFH, and central conducting lymphatic anomaly (CCLA), but the mechanisms underlying these different presentations remain unclear (21, 22, 25, 26, 28). The clinical descriptions of patients with a lymphatic phenotype such as LRFH and CCLA also include clear features of venous disease such as varicose veins, venous hypertension, or venous reflux. Similar to the cases presented, it seems likely that the patients reported by Li et al. may also be affected by VV aplasia and deep venous reflux (considering their increased lower limb pigmentation and venous stasis; ref. 25). The early age at onset of clinical signs of venous insufficiency (for example, varicose veins, hemosiderin deposition) in affected individuals, and the near absence of VVs in the scanned veins of affected children observed here, is consistent with a failure of VV formation, rather than early degeneration. These features are not described for CM-AVM2, or vein of Galen aneurysmal malformation, and it is unclear whether the mutations causing these syndromes will also cause VV defects (22, 26). EphrinB2 is required for normal CV formation in mice, but no CV defects were noted on echocardiography in the patients reported here, or those reported elsewhere (21, 25, 28). It remains unclear how, in the settings of developmental blood vessel formation and in the adult capillary bed, ephrinB2–EphB4 interaction leads to specification and subsequent maintenance of arterial and venous endothelia, yet both are expressed in mature veins to regulate the formation of valves (12, 22, 23, 38, 41). Further work is needed to delineate the context and maturation-dependent regulation of these endothelia.

Previous in vitro analysis of the *EPHB4* mutations studied here (p.Arg739Glu and p.Ile782Ser) demonstrated that they exhibit greatly reduced kinase activity, but do not exert a dominant negative effect on the expression of WT EPHB4 protein (21). Any effect on WT EPHB4 activity is unknown. The ratio of ephrinB2 to EphB4 expression is disturbed at both mRNA and protein level in ECs cultured from patient arteriovenous malformations, with greatly reduced EphB4 expression compared with a control cell line (56). The mutant EPHB4 protein implicated in CM-AVM2 becomes trapped in vesicles (22, 28), whereas that implicated in LRFH is presented on the cell membrane (28), but the exact signaling implications of these findings are yet to be elucidated. The requirement for ephrin/Eph signaling at multiple stages of VV development and maintenance complicates any attempt to develop molecular therapy aiming to directly restore valve function. It is possible that pharmacological stimulation or inhibition of the pathway downstream of EPHB4 might be helpful to overcome the resulting aberrant signaling (22, 25)

The extent of overlap of the genetic causes of VV failure and varicose veins is unclear since regulation of VVs is understudied, but some important indications of similarity have already emerged, including the identification of *PPP3R1* and *PIEZO1* in genome-wide association studies of varicose veins, and in mice as critical regulators of VV development (11, 57, 58). Delineating the roles of the various genes implicated in VV pathogenesis is important and may lead to novel therapies, which could be targeted toward patients at risk of deterioration to chronic ulceration (59)

In this study we have identified a striking “boundary” in the endothelial expression of *Efnb2* at the site of developing VVs (meaning a demarcation between ephrinB2^lo^ upstream cells and ephrinB2^hi^ VFCs and cells immediately downstream), and that both ephrinB2 and EphB4 are required for normal organization of VFCs at this critical stage of development in mice. Because ephrinB2 remains the only known ligand for EphB4, this leads us to speculate that an ephrinB2–EphB4 interaction within venous endothelia regulates VV formation. We also show that EphB4 is required for VV maturation. On E18, in areas where VFCs appeared to be in the process of reorientating to become transversely aligned, EphB4 expression was stronger just upstream of the ephrinB2-expressing VFCs. We speculate that at this time point, EphB4^hi^ regions upstream from VFCs may be acting to repel ephrinB2^hi^ VFCs, guiding them to reorientate to lie transversely across the vessel to form a line across the anterior of the lumen. We were unable to localize ephrinB2 because of a lack of specific antibodies, and this inability to colocalize EphB4 and ephrinB2 is a limitation of our study. In WT littermates VFC polarity was aligned with the boundary and developing ring of VFCs, whereas after *Efnb2* deletion, VFC polarity was disorganized and cells were spread over a wider upstream–downstream region. These results are consistent with previous in vitro findings, showing that the ephrinB2–EphB4 interaction leads to separation and clustering of initially mixed populations of EphB4-expressing and ephrinB2-expressing ECs (47). In vitro, treatment with ephrinB2-Fc stimulates migration of HUVECS, and it is possible that ephrinB2 promotes the migration of VFCs (60).

It remains unknown how *Efnb2* expression within veins is regulated. We have shown that the *Efnb2* boundary forms as the VFCs organize, and it may be regulated by the VFCs themselves as they organize. Notably, BMP9 controls lymphatic remodeling and LV formation, and induces *Efnb2* expression in lymphatic and blood endothelia in vitro, but it is not known whether there is a VV phenotype in *Bmp9^–/–^* mice (61, 62). The extent to which there is proliferation of VFCs between E18 and P0, or whether there is de novo differentiation of new Prox1^hi^ cells from surrounding endothelium, remains unclear.

Normal blood flow is required for postnatal VV maturation (11), and *Efnb2*-dependent protrusion of cells into the lumen on P0 could expose VFCs to higher fluid shear forces, particularly as the vessel lumen becomes more acutely narrowed (e.g., at stage 2 of VV development; ref. 12). Shear-regulated signaling might coordinate subsequent events in VV formation, for example, commissure formation. In embryonic stem cell–derived ECs in vitro, *Efnb2* is upregulated by shear stress, which may contribute to the stimulation of VV leaflet growth postnatally (12, 63). This notion is consistent with the role of the oscillatory shear stress/Gata2/Foxc2 axis in LV endothelial differentiation, and the potential role of wall shear stress gradients in demarcating the locations of valve formation upstream of tributaries (64–66). Deletion of the mechanosensory ion channel *Piezo1* results in defective VVs on P3, again consistent with a role for fluid shear in patterning VV (in addition to LV) formation (57, 67).

Signaling downstream of ephrin–Eph interactions can, for example, inhibit gap junction formation at the boundary between 2 cell populations, likely by cell repulsion preventing stable contacts between cells (32). It seems likely that cell-cell repulsion between ephrinB2^hi^ VFCs and EphB4^hi^;ephrinB2^lo^ upstream cells on E18 patterns the migration of VFCs. It is unknown whether gap/junction signaling is important in this process, but loss of either Cx37 or Cx43 in mice leads to a similar phenotype with failure of VFC organization (11). Homozygous *Efnb2* deletion disrupted the normally highly restricted expression patterns of Cx37 and Cx43 on P0, suggesting that gap junctional communication is disrupted. Gap junction plaque size varies depending on how many channels are clustered in the plaque. It is possible that plaques were present but much smaller, although this would also be expected to reduce cell-cell communication (68). We were unfortunately unable to develop experiments to demonstrate gap junctional VFC cell-cell communication in vivo, or confirm how this may be disrupted after deletion of *Ephb4* or *Efnb2*.

Mutations in *EFNB1* cause craniofrontonasal syndrome, whereas mice heterozygous for *Efnb1* display skull defects that are thought to be mediated by inhibition of normal gap junctional communication via Cx43 at ectopic ephrin-Eph boundaries. EphrinB1 directly interacts with Cx43 and regulates its cellular distribution, and disruption of gap junction plaques was seen in *Efnb1^+/–^* mice (46). Although deletion of *Efnb2* resulted in loss of large Cx37 plaques in VFCs, any direct interaction between ephrinB2 and Cx37 remains to be determined. Although not directly demonstrated in our experiments, it is reasonable to assume that after *Efnb2* deletion, as the Prox1^hi^ VFCs are further apart and are physically separated, there will be less communication between these cells via gap junctions (e.g., incorporating Cx37). EphrinB2 organizes VFC positioning and therefore facilitates the formation of functional gap junctions between adjacent VFCs. It is plausible, therefore, that disruption of connexin expression patterning and gap junctional communication may be part of the mechanism that underlies the phenotype seen after *Efnb2* deletion (46).

In WT mice on P0, Cx43 was expressed upstream of the developing VV and was not clearly expressed by the Prox1^hi^ VFCs that express ephrinB2 (11). Cx43 is clearly expressed by cells that also express EphB4. With deletion of *Efnb2,* Cx43 expression appeared more dispersed throughout the femoral vein, suggesting ephrinB2 is required for the restriction of the Cx43 expression domain. In cardiomyocytes, EphB4 physically associates with Cx43, and EphB activation inhibited cardiomyocyte gap junctional electrical coupling (69). It is possible that in upstream endothelia, signaling through EphB4 could inhibit gap junction communication via Cx43.

It is unclear why the VV phenotype after *Ephb4* deletion was slightly weaker than that in *Efnb2* deleted mice. EphrinB2 is more promiscuous, binding to EphB4, EphB3, and EphB2, whereas EphB4 exclusively interacts with ephrinB2 (38, 70). Isolated knockout of either *Ephb2* or *Ephb3* does not induce any cardiovascular phenotype, but a third of double knockouts have severely defective angiogenesis that resembles much of the phenotype of *Efnb2^–/–^* mice (38). EphB3 expression has been reported in veins (whereas EphB2 is expressed in nonvascular mesenchymal cells), but we could not detect specific signals for EphB2 or EphB3 in veins by immunohistology (data not shown). EphrinB2 regulates cell morphology and motility independently of binding its receptors in vitro, which could partly explain the stronger phenotype seen with *Efnb2* deletion (71). In sprouting angiogenesis, ephrinB2 is required for endocytosis and signaling of other important regulators of EC function including Vegfr2 and Vegfr3 (which are expressed in developing VVs), and could play similar roles in VFC organization (12, 39, 40). The slight difference in the phenotypes after deletion of *Efnb2* and *Ephb4* could be caused by differences in their protein stability, which we were unable to investigate, in part because of the lack of specific antibodies raised against ephrinB2. We could not confirm reduced *Ephb4* or *Efnb2* mRNA levels after conditional gene deletion, due to our inability to specifically isolate VV cells, but this has been confirmed for *Efnb2* deletion in lymphatic endothelium (72).

Detachment of VFCs from their underlying basement membrane has previously been identified in LV formation, during angiogenesis, and we now show it here in VV formation (48, 73). Due to detachment, cell-cell contacts are highly restricted, and this is likely to impact cell/cell signaling processes (48, 74). In vitro, soluble ephrinB2-Fc acts antiadhesively, and the high ephrinB2 expression in VFCs could promote their detachment from the underlying basement membrane to facilitate reorientation and organization (47).

We have previously analyzed VFC nuclear reorientation and elongation in wholemount confocal microscopy to characterize phenotypes on P0/stage 1 of VV development (11). Here, we show that VFCs not only protruded into the vessel lumen at this stage, but that this protrusion was abolished after homozygous *Efnb2* deletion. We also identify that ingress of interstitial cells was already occurring at this early stage and confirm their persistence in P6 and adult murine VV, and in adult human VV. Their existence has previously been demonstrated in human, rat, and rabbit VV, in contrast to LV, which lack interstitial cells (2, 49, 54). In lymphovenous valve development, mural cells are recruited into the valve leaflets during maturation, but the developmental origin of these cells in VVs is currently unknown (54). The identity, origin and functions of these cells in VVs will be the subject of future studies.

Our data showing that EphB4 was required for postnatal development is consistent with the phenotype resulting from *Efnb2* deletion on P2 or P0 (11, 12). Almost all Prox1^hi^ and Foxc2^hi^ VFCs were absent on P6, in contrast to deletion of *Ppp3r1* (CnB1), in which a clear ring of Prox1- and Foxc2-expressing cells remains (11). This is consistent with a requirement for EphB4 (and ephrinB2) to develop/maintain the phenotype of free-edge cells to P6, rather than just growth of VV leaflets (11). The failure to establish a local reduction in the density of SMCs around the VV on P6 after *Ephb4* deletion on P0 is consistent with the endothelial VFC/SMC signaling that controls this reduction in SMC density around LVs (31, 75–77).

### Conclusions.

In addition to an increased risk of lymphatic-related fetal hydrops, we show that patients carrying heterozygous mutations in *EPHB4* had very few VVs, with early onset deep venous reflux indicating that the observed venous insufficiency was due to VV aplasia. By studying mice, it was demonstrated that ephrinB2 and EphB4 patterned the organization of VFCs on P0 and was required for cellular reorientation, elongation, protrusion, and proliferation, adding to our understanding of the complex VV developmental program. Postnatal deletion of *Ephb4* led to complete loss of the valve, which could explain the phenotype observed in the patients.

## Methods

### Human VV ultrasonography.

The brachial, basilic, popliteal, and short saphenous veins underwent ultrasonographic evaluation in London (Phillips IU22 with L17-5 MHz/L9-3 MHz probes) and VV maximum leaflet measurements obtained offline (Xcelera Cath Lab software, Phillips). Reproducibility was determined previously (11). For each vein, the number of VVs and VV length were normalized to the mean value in the respective control veins from our existing control population and additional new controls, and the mean number of VVs per vein, per patient, was compared. Deep venous (popliteal) reflux duration was measured bilaterally after distal manual compression while standing, and the mean was taken, with reflux defined as 0.5 second or longer and severe reflux as longer than 1 second (78–80). Because deep venous reflux is rare, popliteal venous reflux was not routinely measured in the entire control population, but was subsequently measured in additional controls (81). Genotyping was performed at St George’s, University of London and in Bergen (21).

### Mouse lines.

WT analyses were carried out in BALB/C mice obtained from Charles River UK. *Prox1CreER^T2^* (12), *Rosa26^mTmG^* (82), *Efnb2^lx^* (83), and *Efnb2^GFP^* (84) mice have been previously described and were maintained on C57BL/6 backgrounds. Tamoxifen/4OH-tamoxifen (in peanut or sunflower oil, MilliporeSigma) was injected i.p. either 1 mg on E15 for analysis on P0, or 50 μg on P0 for analysis on P6 in order to induce Cre activity in *Prox1CreER^T2^* mice (12). To delay labor, 37.5 μg/g.Ms weight progesterone was given i.p. on E15 and E18 and embryos were analyzed on “E19,” equivalent to P0. We compared VV in *Prox1CreER^T2+^* with *Prox1CreER^T2–^* littermate controls in all deletion experiments.

### Electron microscopy.

Mice were culled and perfused via the aorta with heparinized PBS (hPBS, 25 mg/L; MP Biomedicals) prior to fixation overnight in glutaraldehyde (2.5% v/v in 0.1 M cacodylate buffer, pH 7.4, 4°C) and postfixation in osmium tetroxide (1% w/v in 0.1 M cacodylate, pH 7.4, 4°C) for 1.5 hours. All samples were dehydrated through graded ethanols, equilibrated with propylene oxide, infiltrated with epoxy resin (TAAB), and polymerized at 70°C for 24 hours. Semithin sections (0.45 μm) were cut and stained with 1% Toluidine Blue. For analysis of protruding VFCs, more than 90 serial semithin sections were analyzed per sample (2-tailed unpaired *t* test). For 3D reconstructions on P0, semithin sections (0.45 μm) were photographed (Leitz DMRB microscope, Micropublisher 3.3RTV camera), aligned in NIH ImageJ and reconstructed using Amira (Thermo Fisher Scientific). Ultrathin sections (50–70 nm, Reichert-Jung ultramicrotome) were mounted and contrasted using uranyl acetate/lead citrate for examination (Hitachi H7600, 80kV, AMT digital camera; ref. 85). For quantification of interstitial cells on P6, the length of the leaflet was measured in NIH ImageJ, and the number of whole interstitial cell nuclei was counted. Human great saphenous veins (obtained during coronary artery bypass grafting) were opened prior to processing as per murine samples, with visualization of ultrathin sections using a Hitachi S-3500N microscope.

### IHC.

Mice were culled and perfusion fixed via the aorta and femoral vein by perfusion with hPBS followed by 4% formaldehyde and then further fixed for 24 hours. The external iliac and femoral veins were excised and embedded in wax, and 5-μm sections were incubated with primary antibody and washed prior to amplification using polymer horseradish peroxidase (Menarini) and signal detection using SG peroxidase substrate (Vector). Sections were photographed using a Micropublisher 3.3RTV camera mounted on a Leitz DMRB microscope with PL Fluotar ×20 lens (Leica). For human Connexin IHC, see Supplemental Methods.

### Wholemount immunostaining and analysis.

Mice were culled and perfused with hPBS via the aorta prior to fixation in 4% paraformaldehyde followed by blocking in 3% v/v donkey serum, 0.3% Triton X-100, and further dissection prior to incubation with primary antibodies, and washing prior to localization with fluorophore-conjugated secondary antibodies. Samples were finally dissected and mounted in Prolong Gold (Invitrogen). Valves were imaged using a Leica SP5 confocal microscope (1024 × 1024 resolution, 8-bit) to produce Z projections (NIH ImageJ) of median filtered (Leica LASAF/ImageJ, except for connexin localization or fluorescence quantification) stacks. Lookup tables were linear. Control samples were incubated with either the appropriate nonimmune IgG and then secondary antibody or streptavidin-conjugated fluorophore alone (Supplemental Figure 2A).

For analysis of VFC organization, Prox1^hi^ nuclear elongation (proportion with length/width ratio greater than or equal to 2) and reorientation (proportion with long axis greater than or equal to 40° from the vessel center line, in nuclei with length/width ratio greater than or equal to 2) were quantified in *Z* projections (NIH ImageJ) as previously described (11, 48).

For analysis of *Efnb2^GFP^* expression on E18-P0 (Figure 4), *Z* projections of confocal *Z* stacks were oriented with flow left to right and the center line of the vessel horizontal. For each valve, 7–12 10 × 100–μm regions of interest, each centered on the VFC upstream edge, were analyzed (NIH ImageJ). Mean intensity profiles for each fluorophore were converted to *Z* scores and the mean of 6 VVs was plotted. On E18, areas with and without Prox1^hi^ organizing VFCs were analyzed separately.

For analysis of areas of expression of Ephb4 and Efnb2-GFP on E18 (Figure 2B), an XZ projection (13.6-μm deep) across the reorientating VFCs was reconstructed (NIH ImageJ) and the relative fluorescence intensity profile for Efnb2-GFP and EphB4 was plotted. For quantification, for each valve 4–6 50-μm linear regions of interest were drawn, centered on the VFC leading edge, on E18, for *n* = 6 VVs. Ephb4 upstream versus downstream intensity was compared (2-tailed *t* test).

For analysis of cell orientation by coimmunostaining of nucleus and Golgi, stacks of 0.5-μm optical sections were analyzed (NIH ImageJ) to identify the Golgi for each VFC, and an arrow was drawn from nuclear center to Golgi center. The *Z* projection of all arrows is shown.

### Antibodies.

The following antibodies were used: rabbit anti-Cx43 (Cell Signaling Technology 3512), Cx37 (CX37A11, Alpha Diagnostics), Prox1 (11-002P, Angiobio), ki67 (ab15580, Abcam), and Golgi apparatus protein 1 (ab103439, Abcam); sheep anti-Foxc2 (AF6989, R&D); goat anti-EphB4 (BAF446, R&D); rat anti-PECAM1 (clone MEC 13.3, BD); and mouse anti-α smooth muscle actin (clone 1A4 conjugated to Cy3, MilliporeSigma). For fluorescence signal detection, secondary antibodies or streptavidin were conjugated to Dylight-405/488/550/649 (Jackson ImmunoResearch).

### Statistics.

For VV developmental stage 0–4 quantification, data represent the proportion of VV reaching each developmental stage. *P* values represent the difference in proportion of valves at each stage versus their WT littermates (χ^2^/Fisher’s exact test as appropriate). Comparisons of VFC nuclear elongation and reorientation between groups were analyzed by 1-way ANOVA and Bonferroni’s post hoc tests. Age and sex matching for patient ultrasonography was tested respectively by 2-tailed unpaired *t* test and Fisher’s exact test. All analyses were carried out using IBM SPSS Statistics 24, and Graphpad PRISM v8. A *P* value of less than 0.05 was considered significant. Ultrasonographers were blinded to participant genotype during scanning and image analysis/quantification. Experiments were not randomized.

### Study approval.

All human studies and animal studies were carried out in accordance with national regulations and ethical approvals in the United Kingdom and Sweden (Health Research Authority 12/LO/1164, 10/H0701/68, C130/15). Written informed consent was obtained from all participants.

## Author contributions

OL, JW, CS, MI, AK, AA, MHV, MF, GVB, RAF, ASP, SP, PM, SJ, SB, SM, PO, TM, BM, PS, and AS designed research studies. OL, JW, CS, MI, AA, MHV, MF, AK, SP, and GVB conducted the experiments and acquired and analyzed data. PM, SJ, SB, SM, and PO provided the patients. OL, AS, and TM funded this study. OL, JW, CS, MI, AK, AA, MHV, MF, GVB, RAF, ASP, SP, PM, SJ, SB, SM, PO, TM, BM, PS, and AS reviewed drafts and approved the manuscript.

## Supplementary Material

Supplemental data

## Version 1 08/17/2021

In-Press Preview

## Version 2 09/22/2021

Electronic publication
