# Supplementary material for: Mutations in EPHB4 cause human venous valve aplasia
Source: JCI Insight. 2021 Sep 22;6(18):e140952. doi: 10.1172/jci.insight.140952 (PMC8492339; doi:10.1172/jci.insight.140952)
Supplement: Supplemental data [file jciinsight-6-140952-s177.pdf]

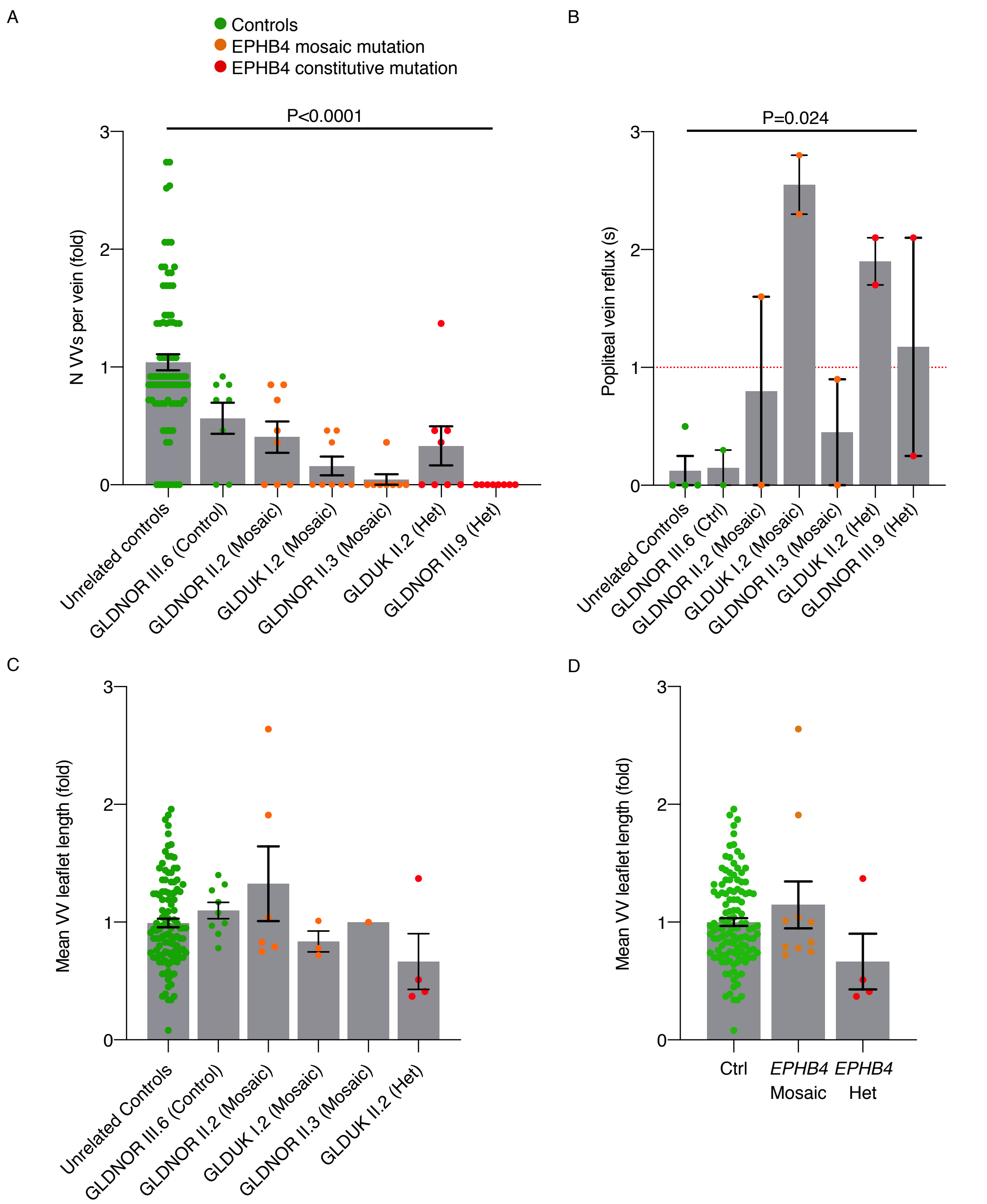

A
Controls
EPHB4 mosaic mutation
EPHB4 constitutive mutation
P<0.0001
N VVs per vein (fold)
0
1
2
3
Unrelated controls
GLDNOR III.6 (Control)
GLDNOR II.2 (Mosaic)
GLDUK I.2 (Mosaic)
GLDNOR II.3 (Mosaic)
GLDUK II.2 (Het)
GLDNOR III.9 (Het)
B
P=0.024
Popliteal vein reflux (s)
0
1
2
3
Unrelated Controls
GLDNOR III.6 (Ctrl)
GLDNOR II.2 (Mosaic)
GLDUK I.2 (Mosaic)
GLDNOR II.3 (Mosaic)
GLDUK II.2 (Het)
GLDNOR III.9 (Het)
C
Mean VV leaflet length (fold)
0
1
2
3
Unrelated Controls
GLDNOR III.6 (Control)
GLDNOR II.2 (Mosaic)
GLDUK I.2 (Mosaic)
GLDNOR II.3 (Mosaic)
GLDUK II.2 (Het)
D
Mean VV leaflet length (fold)
0
1
2
3
Ctrl
EPHB4 Mosaic
EPHB4 Het

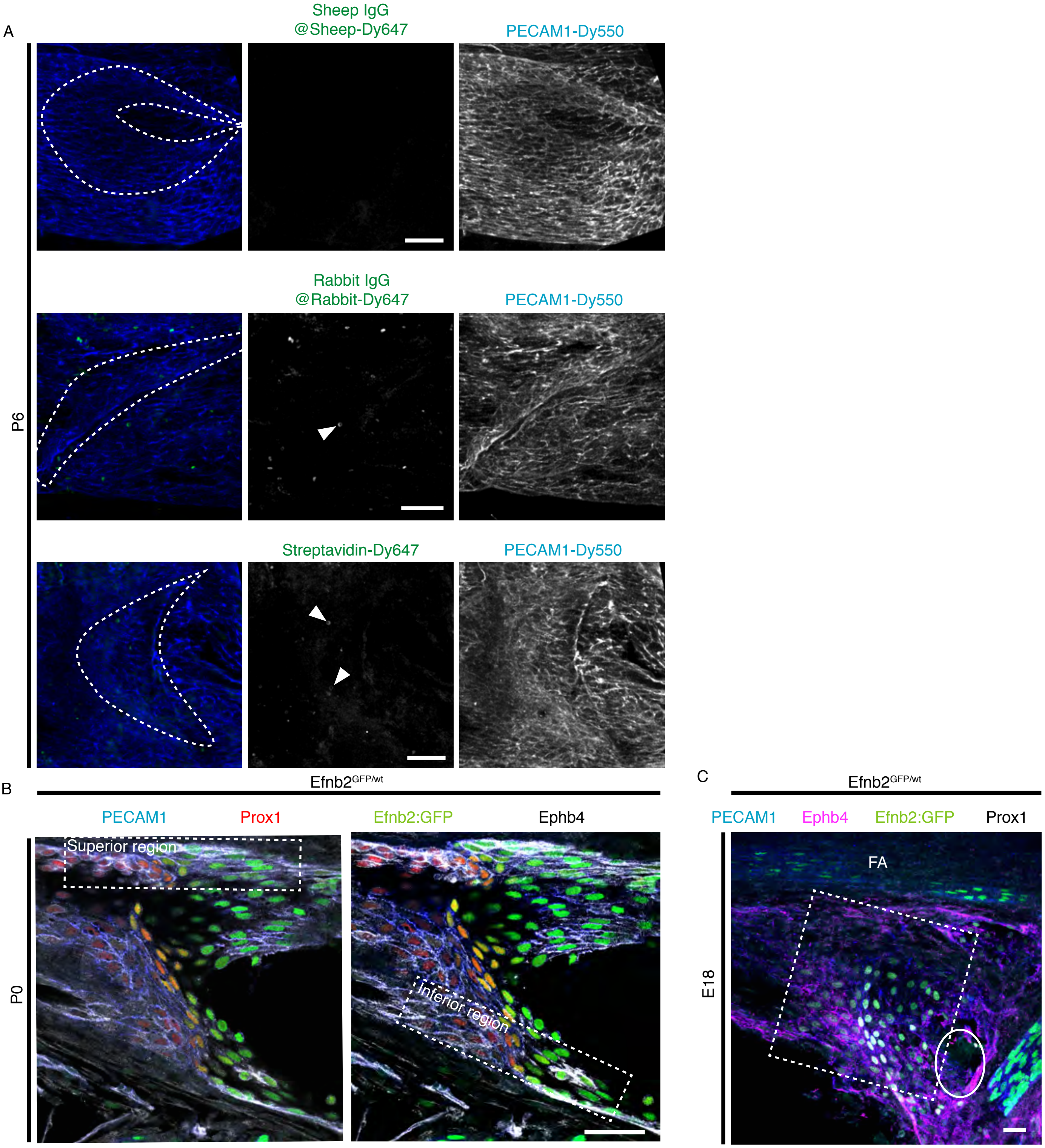
A
Sheep IgG
@Sheep-Dy647
PECAM1-Dy550
Rabbit IgG
@Rabbit-Dy647
PECAM1-Dy550
Streptavidin-Dy647
PECAM1-Dy550
P6
B
Efnb2GFP/wt
PECAM1
Prox1
Efnb2:GFP
Ephb4
Superior region
Inferior region
P0
C
Efnb2GFP/wt
PECAM1
Ephb4
Efnb2:GFP
Prox1
FA
E18

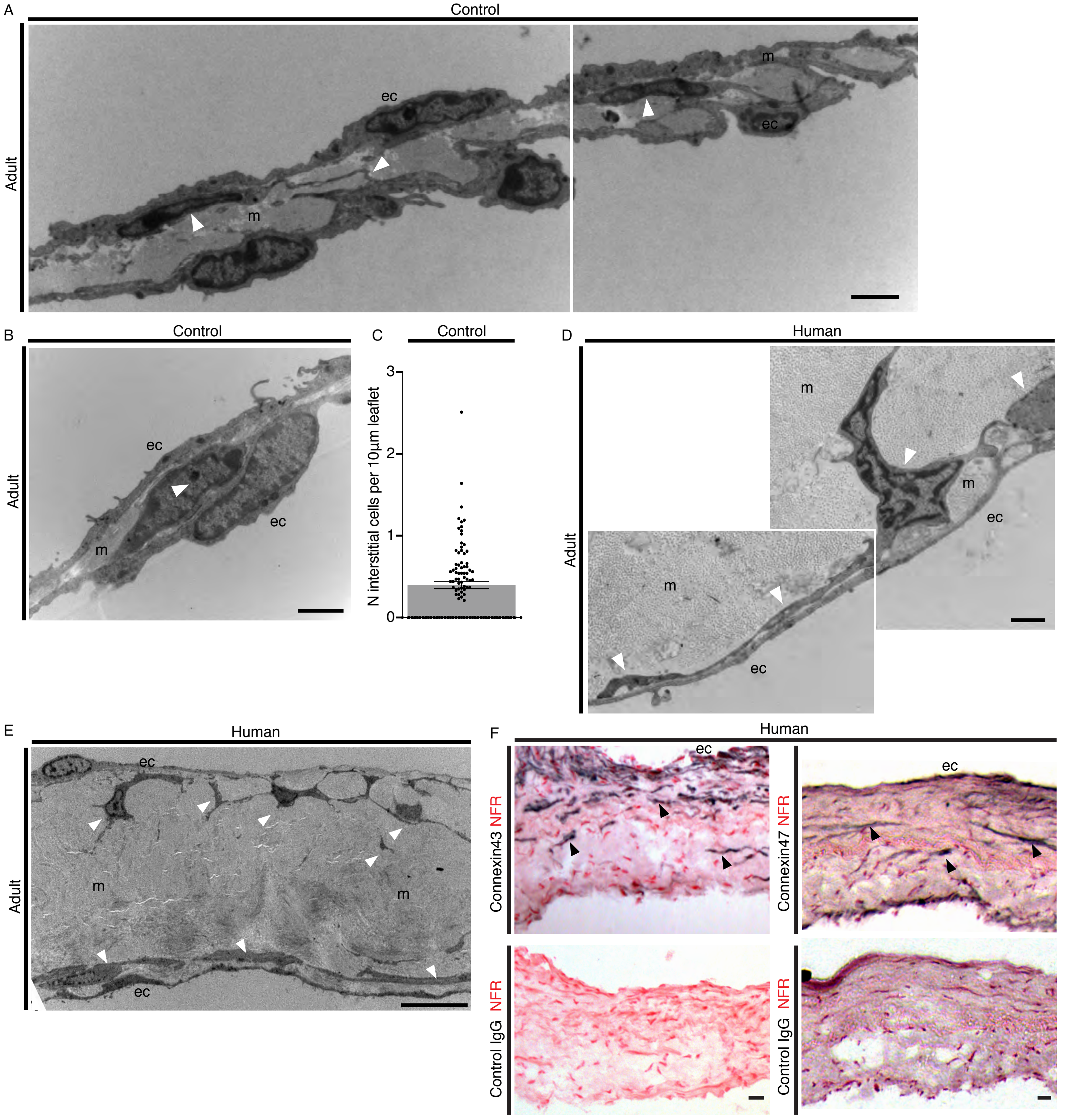
A
Control
Adult
ec
m
m
ec
B
Control
Adult
ec
ec
m
C
Control
N interstitial cells per 10µm leaflet
3
2
1
0
D
Human
Adult
m
m
ec
m
ec
E
Human
Adult
ec
m
m
ec
F
Human
ec
Connexin43 NFR
ec
Connexin47 NFR
Control IgG NFR
Control IgG NFR

**Supplementary Table 1: Study participants**

| Family | Group | Sex, Age | Clinical History/examination |
|---|---|---|---|
| Unrelated controls | | M 25 | No varicose veins |
| | | F 30 | |
| | | F 24 | |
| | | M 24 | |
| | | M 37 | |
| | | F 42 | |
| | | F 42 | |
| | | F 53 | |
| | | F 58 | |
| | | M 69 | |
| | | M 23 | |
| | | M 66 | |
| $GLD_{UK}$ I.2 | Mosaic, 37% in blood | M 56 | Varicose veins, bilateral persistent peripheral lymphedema in the lower limbs since age 15. |
| $GLD_{UK}$ II.2 | Constitutive | F 36 | Varicose veins, no clinical signs of persistent peripheral lymphedema, but lower limb lymphoscintigraphy showed bilateral impaired lymphatic drainage. |
| $GLD_{NOR}$ II.3 | Mosaic, 50% | F 39 | Varicose veins since late teens (operated). No clinical signs of persistent peripheral lymphedema, but lower limb lymphoscintigraphy showed bilateral impaired lymphatic drainage. |
| $GLD_{NOR}$ III.9 | Constitutive | M 7 | Mildly prominent (but not varicose) veins on posterior leg. No clinical signs of peripheral lymphedema. |
| $GLD_{NOR}$ II.2 | Mosaic, 13-31% | F 39 | Varicose veins since age 23 (operated). No clinical signs of persistent peripheral lymphedema, but lower limb lymphoscintigraphy showed multiple tortuous lymphatic tracts. |
| $GLD_{NOR}$ III.6 | Unaffected relative (control) | M 9 | Normal |

## Supplementary Table 1 Legend

Family identifiers refer to Refs (1, 2). The *EPHB4* (NM_004444.4) mutation in $GLD_{UK}$ is c.2216G>A, p.Arg739Glu, and in $GLD_{NOR}$ it is c.2345T>G, p.Ile782Ser. $GLD_{NOR}$ II.2 and II.3 are monozygotic twins and mosaic carriers of the *EPHB4* variant; $GLD_{NOR}$ II.3 was almost 50:50 wildtype to mutant variant (i.e. similar to a heterozygous constitutive carrier) in most tissues, but $GLD_{NOR}$ II.2 had a lower mutation load (13-31% in the tissues measured) and an associated milder VV phenotype (i.e. higher mean number of valves than $GLD_{NOR}$ II.3, Fig. 1B).(1) $GLD_{UK}$ I.2 was previously identified as a constitutive mutation carrier, but has since been confirmed to be mosaic, with approximately 37% mutation load in his blood.(2) Clinical history related to the venous and lymphatic phenotype is shown here, for a detailed clinical history the reader is referred to references.(1, 2)

## Supplementary Methods

*Human VV connexin histology*:

For localization of connexins in human VVs (obtained from patients undergoing coronary artery bypass grafting) 10µm frozen sections were thawed and fixed in -$20^{o}$c acetone prior to quenching of endogenous peroxidase using 3%$H_20_2$, blocking (X0909, DAKO), incubation with primary antibodies, and amplification (MP-XCP, Menarini) according to the manufacturer's instructions. Signal detection was with alkaline phosphatase (DAKO), and the counterstain was Nuclear Fast Red (Vector). Sections were photographed using a Micropublisher 3.3RTV camera mounted on a Leitz DMRB microscope. Primary antibodies were raised in rabbit to CX43 (Invitrogen 71-0700), CX47 (Sigma SAB2100924). Controls were incubated with non-immune rabbit IgG (R&D)

## Supplementary References

1. Martin-Almedina S, et al. EPHB4 kinase-inactivating mutations cause autosomal dominant lymphatic-related hydrops fetalis. *J Clin Invest.* 2016;126(8):3080-8.
2. Martin-Almedina S, et al. Janus-faced EPHB4-associated disorders: novel pathogenic variants and unreported intrafamilial overlapping phenotypes. *Genetics in medicine : official journal of the American College of Medical Genetics.* 2021.

## Supplementary Figure Legends

### Supplementary Figure 1

A) The mean number of VVs per vein is shown for the unrelated controls, and for each participant from the affected families. Each data point indicates a single vein (8 veins per participant). Mosaic carriers are arranged in order of approximate *EPHB4* mutation load, see Supplementary Table 1. (P<0.0001, ANOVA)

B) The mean duration of reflux is shown for each participant from the affected families. Each data point represents the left or right popliteal vein in that individual. A reflux duration >1s indicates severe deep venous reflux. A single functioning valve near the analysed venous segment may prevent reflux, which may explain why some veins in affected individuals did not exhibit reflux, despite significant reductions in the overall number of valves. Deep venous reflux was not assessed in all of the unrelated control population because it is rare and reference values are well established. (P=0.024, ANOVA)

C) The mean VV leaflet lengths are shown, for those VVs detected, for each participant and also, in (D), by genotype. No VVs were detected in GLDNORIII.9. P=ns for C and D. Data points represent individual valves.

Green = control, Orange = mosaic for *EPHB4* mutations, Red = heterozygous for *EPHB4* mutation. Error bars indicate sem.

**Supplementary Figure 2**

A) Isotype immunofluorescence staining controls, using the indicated fluorophore-conjugated secondary antibodies, following incubation of samples with the appropriate non-immune IgG (for sheep and rabbit), or without primary antibody (for Streptavidin). The valve is outlined in the combined image (dotted white line). Arrowheads indicate residual autofluorescent erythrocytes in the vein lumen.

B) This figure relates to Fig.2D, and shows uncropped and unrotated images of 6μm z-projections to visualise an approximate single cell layer of the upper and lower regions of a valve. The regions of the valve reproduced in Fig.2D are indicated by dotted boxes.

C) A lower magnification image of Figure 2A (outlined by dotted box). FA = femoral artery. The site of a tributary is circled.

Bars = 20μm

**Supplementary Figure 3**

A) Two adjacent representative TEM micrographs of part of an adult murine control valve leaflet, indicating the distribution of interstitial cells embedded within the matrix core. Endothelial cells are indicated by ‘ec’, extracellular matrix by ‘m’, and interstitial cells embedded in matrix by white arrowheads.

B) A further example of an interstitial cell nucleus embedded in matrix, surrounded by a layer of endothelial cells on each leaflet surface.

C) The mean number of interstitial cells identified per 10μm leaflet length is shown (N=100 TEM sections, from three levels of a VV at P6). Error bars indicate sem. (A-C are wildtype BALB/C mice)

D) Two adjacent TEM micrographs of part of an adult human great saphenous VV leaflet are shown, with the endothelial cell layer to bottom right, and the extensive matrix of the leaflet core to the top left of the images. Multiple morphologies of interstitial cells were identifiable, frequently extending laterally under the endothelial cell layer.

E) A further example of an adult human great saphenous VV leaflet. Images in D-E are representative of multiple sections obtained from four valves. Abbreviations as in A-B.

F) Light micrographs showing Connexin43 and Connexin47 immunolocalised to interstitial cells (arrowheads), as well as endothelial cells (ec) in adult human VV leaflet, but absent immunostain in isotype controls. NFR = Nuclear Fast Red counterstain.

Bars = 2μm in A-B, 1μm in D, 10μm in E, 20μm in F
